# Memory Devices with HfO_2_ Charge-Trapping and TiO_2_ Channel Layers: Fabrication via Remote and Direct Plasma Atomic Layer Deposition and Comparative Performance Evaluation

**DOI:** 10.3390/ma18050948

**Published:** 2025-02-21

**Authors:** Inkook Hwang, Jiwon Kim, Joungho Lee, Yeonwoong Jung, Changbun Yoon

**Affiliations:** 1 Department of Advanced Materials Engineering, Tech University of Korea, Siheung-si 15073, Republic of Korea; 2 Korea Evaluation Institute of Industrial Technology, Seoul 06152, Republic of Korea; 3 Department of Materials Science and Engineering, University of Central Florida, Orlando, FL 32816, USA

**Keywords:** HfO_2_, TiO_2_, remote plasma, direct plasma, charge-trapping memory, oxide semiconductor, plasma damage, atomic layer deposition

## Abstract

With the improvement of integration levels to several nanometers or less, semiconductor leakage current has become an important issue, and oxide-based semiconductors, which have replaced Si-based channel layer semiconductors, have attracted attention. Herein, we fabricated capacitors with a metal–insulator–semiconductor–metal structure using HfO_2_ thin films deposited at 240 °C and TiO_2_ thin films deposited at 300 °C via remote plasma (RP) and direct plasma (DP) atomic layer deposition and analyzed the effects of the charge-trapping and semiconducting properties of these films. Charge-trapping memory (CTM) devices with HfO_2_ (charge-trapping layer) and TiO_2_ (semiconductor) films were fabricated and characterized in terms of their memory properties. Al_2_O_3_ thin films were used as blocking and tunneling layers to prevent the leakage of charges stored in the charge-trapping layer. For the TiO_2_ layer, the heat-treatment temperature was optimized to obtain an anatase phase with optimal semiconductor properties. The memory characteristics of the RP HfO_2_–TiO_2_ CTM devices were superior to those of the DP HfO_2_–TiO_2_ CTM devices. This result was ascribed to the decrease in the extent of damage and contamination observed when the plasma was spaced apart from the deposited HfO_2_ and TiO_2_ layers (i.e., in the case of RP deposition) and the reduction in the concentration of oxygen vacancies at the interface and in the films.

## 1. Introduction

The growing popularity of mobile electronic devices, such as smartphones, laptops, and tablets, has resulted in the increased use of nonvolatile memory. Consequently, considerable attention has been drawn to charge-trapping memory (CTM) devices, such as silicon–oxide–nitride–oxide–silicon flash memory, at nodes below 10 nm [1,2,3,4,5,6]. Compared with conventional floating-gate devices, CTM devices offer several advantages, including a higher reliability, lower operating voltage, simpler manufacturing process, smaller cell size, and lower program/erase (P/E) voltage. However, the decrease in the thicknesses of tunneling oxide and charge-trapping oxide layers hinders the improvement of CTM data retention time and P/E speed [7,8,9,10,11,12], largely because of the trade-off relationship between these parameters. Thin tunnel oxide layers allow one to increase the P/E speed of silicon–oxide–nitride–oxide–silicon memory devices by applying a strong electric field to the semiconductor but pose difficulties in retaining data over extended periods. As a result, CTM devices utilizing high-*k* materials (e.g., HfO_2_, Al_2_O_3_, and ZrO_2_) have been actively developed to overcome this problem [13,14,15,16,17].

Owing to its high dielectric constant, bandgap (5.5–6.0 eV), dielectric breakdown field (3.9–9 MV/cm), trap density, conduction band offset, thermal stability, and durability, HfO_2_ is widely used in electronics, particularly as a transistor dielectric for microprocessors, and is an ideal candidate for semiconductor device [18,19,20,21,22,23] and next-generation flash memory fabrication. Originally, SiO_2_ thin films were employed as high-quality dielectrics because of their low defect density, amorphous structure, and excellent interface properties. However, with the miniaturization of semiconductor devices, SiO_2_ has reached its thickness limit of ~1.0 nm. At such low thicknesses, SiO_2_ films exhibit high leakage currents, which highlights the need for high-*k* materials with the same equivalent oxide thickness but a higher physical thickness [18,19,20,21,22,23,24,25,26,27,28].

Metal oxide semiconductors, such as HfO_2_, TiO_2_, IGZO, and ZnO, have been actively studied as nonvolatile memory components. Oxide MOSFETs have a notably lower off-state leakage current than Si MOSFETs and therefore allow for lower-power memory operation [29,30,31,32,33]. Oxide-based nonvolatile memory typically uses positive and negative gate voltage pulses to achieve program and erase states, respectively. To ensure stable device operation and a long-term reliability of >10 years, one should set a sufficient memory window (difference between programming and erasing voltages) and thus secure an adequate read voltage margin. In CTM, a wide memory window can be achieved by creating more defect sites to trap additional charges [34,35,36,37,38].

Atomic layer deposition (ALD) is the main process for depositing uniform thin films on memory devices with thicknesses of several nanometers and complex trench structures and can be classified as thermal or plasma-enhanced (PE) based on the energy transfer method used for reactive gas activation [39,40,41,42,43,44]. PE ALD is primarily used for semiconductor deposition because of its lower process temperature, higher film density, and faster deposition rate compared with those of thermal ALD and can be further divided into direct plasma (DP) and remote plasma (RP) ALD. Despite the excellent deposition rate and film quality achieved by DP ALD, where plasma is discharged in the process chamber to deliver energy to the reactive gases, the ions in the plasma can collide with the substrate or film surface to damage the interface and degrade the film’s properties. Hence, DP ALD is challenging to apply to trench structures with high aspect ratios [45,46,47]. Moreover, as semiconductor structures have evolved to trench structures with hundreds of laterally stacked layers, as in DRAM, the use of DP ALD, which is characterized by strong directional electric fields and moving radicals, has become impractical. As a result, thermal ALD is still predominantly used despite its low productivity. RP ALD, which is being actively researched as an alternative, addresses plasma damage- and directional field-related issues by separating the plasma discharge area from the process chamber and injecting only activated radicals into the process chamber. However, the limited lifetime of plasma-activated radicals necessitates the optimization of equipment structure and process conditions to realize thin films with the desired properties [48,49,50,51].

Herein, we used RP and DP ALD to fabricate Si^n++^/HfO_2_/Al_2_O_3_/TiO_2_/Al (MHOTM) structures with HfO_2_ and Al_2_O_3_ thin films acting as charge-trapping and tunneling layers, respectively, and compared the characteristics of the corresponding CTM devices. Prior to semiconductor device fabrication, the deposition and annealing temperatures were optimized to enhance the electrical properties of TiO_2_ thin films and produce the anatase phase of TiO_2_. Compared with those fabricated using DP ALD, the devices fabricated using RP ALD exhibited a markedly higher charge-trapping efficiency and larger memory window (3.5 V vs. 2.5 V at an operating voltage of ±7 V) while featuring reliability characteristics equivalent to 10 years. This finding suggests that although RP ALD may introduce intrinsic defects (such as oxygen vacancies) into HfO_2_, the amount of interface defects is minimal, as confirmed by the comparative instrumental analyses of thin films deposited by RP and DP ALD [52,53,54,55].

## 2. Materials and Methods

### 2.1. Fabrication of MHOTM Semiconductor Devices

**Figure 1:**
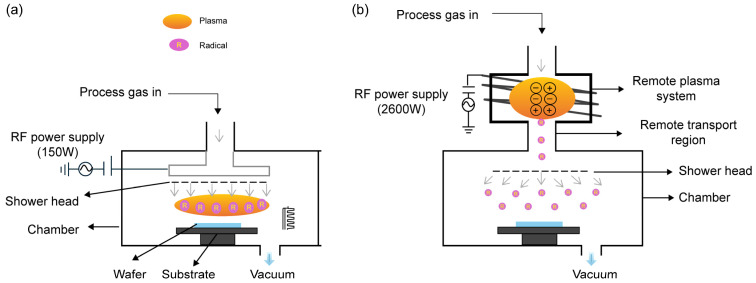
Configurations of (**a**) direct plasma (DP) and (**b**) remote plasma (RP) atomic layer deposition (ALD) systems.

**Figure 2:**
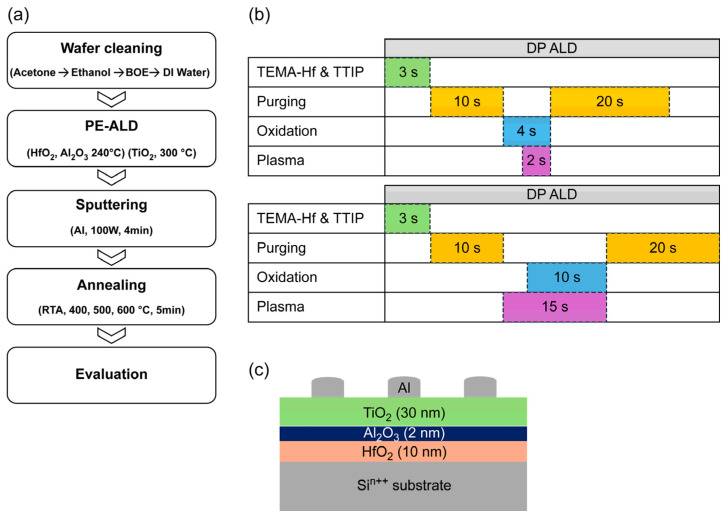
Schematics of (**a**) Si^n++^/HfO_2_/Al_2_O_3_/TiO_2_/Al (MHOTM) device production, (**b**) ALD valve sequences and exposure times utilized for RP and DP processes, and (**c**) MHOTM device structure.

Figure 1a,b show the configurations of DP and RP ALD systems, respectively. In the case of DP ALD, plasma directly contacts the substrate during discharge, whereas in the case of RP ALD, plasma is generated by an inductively coupled plasma method using a plasma–substrate gap and is delivered by an argon (Ar) carrier gas. Herein, the ALD setup was modified to enable the operation of both systems within a single apparatus and thus compare plasma characteristics while eliminating other variables, such as differences in chamber design, gas flow dynamics, and precursor delivery conditions. In the RP mode, only neutral plasma radicals can pass through the mesh, whereas in the DP mode, all plasma radicals can interact with the substrate. Figure 2a,b illustrate the ALD process and optimized valve sequences/exposure times, respectively. For both DP and RP ALD, the conditions were optimized to achieve HfO_2_ and TiO_2_ thicknesses of 10 and 30 nm, respectively. DP ALD was performed using an oxygen injection time of 4 s and a plasma generation time of 2 s, whereas the corresponding values for RP ALD were 10 and 15 s, respectively. Owing to its conductivity (resistivity = 0.0025 Ω cm), a four-inch n^++^-type (100) Si wafer was used as the substrate and bottom electrode. The substrate was cleaned using SC-1 cleaning and immersed into an HF-containing etchant for ~30 s to remove the native oxide layer.

HfO_2_, TiO_2_, and Al_2_O_3_ thin films were deposited on the etched wafer using a DP and RP ALD system (iOV-dx2, iSAC Research, Daejeon, Republic of Korea). Tetrakis (ethylmethylamido) hafnium (iChems, Hwaseong, Republic of Korea), trimethylaluminum (iChems), and titanium tetraisopropoxide (iChems) were used as HfO_2_, Al_2_O_3_, and TiO_2_ precursors, respectively. HfO_2_ was used as a charge-trapping layer (CTL), TiO_2_ as a semiconductor channel layer, and Al_2_O_3_ as a blocking and tunneling layer. DP was generated by a built-in plasma generator with a power of 150 W using O_2_ as the reactive gas. RP was produced at a power of 2600 W (En2ra-RPS, EN2CORE Technology, Daejeon, Republic of Korea). After ALD, Al electrodes with a diameter of 200 μm and thickness of 50 nm were formed using the shadow mask method. Al deposition was conducted at room temperature using direct-current magnetron sputtering (BLS, Gyeonggi-do, Republic of Korea) at 100 W for 4 min. Postmetallization treatment corresponded to rapid thermal annealing (RTA700, PyroTec, Gyeonggi, Republic of Korea) for 5 min under N_2_ at 400, 500, or 600 °C. The structure of the fabricated charge-trapping device is illustrated in Figure 2c.

### 2.2. Structural and Electrical Property Evaluation of Semiconductor Devices

The thickness of the HfO_2_ and TiO_2_ thin films was measured using spectroscopic ellipsometry (Elli-SE-U, Ellipso Technology, Suwon-si, Republic of Korea). The effect of annealing temperature on the crystal structure of the TiO_2_ layer was analyzed using X-ray diffraction (SmartLab, Tokyo, Japan). Transmission electron microscopy (JEM-2100F, JEOL, Tokyo, Japan) was used to probe the structure of the MHOTM devices and measure the lattice spacing and crystallographic orientation of the TiO_2_ layer. High-resolution TEM (HR-TEM) images were obtained to analyze the atomic arrangement and interfacial characteristics between the TiO_2_ layer and adjacent layers. The compositions and defects of the HfO_2_ and TiO_2_ thin films deposited by DP and RP ALD (hereinafter referred to as DP and RP MO_2_ (M = Hf, Ti), respectively) were characterized using X-ray photoelectron spectroscopy (NEXSA, Thermo Fisher Scientific, OR, USA). High-resolution XPS spectra were collected to analyze the oxidation states and chemical bonding environments of Hf and Ti in the thin films. XPS measurements were performed using a monochromatic Al Kα source (hν = 1486.6 eV) with an energy resolution of 1.00 eV and a pass energy of 200.0 eV. An ion gun (1000 eV) and a 200 μm X-ray spot size were used. Background subtraction (Shirley method) and spectral fitting (asymmetric Gaussian–Lorentzian) were conducted using CasaXPS, with calibration applied for accurate binding energy assignment. The surface roughness and crystalline particles of the DP and RP HfO_2_ films were observed using atomic force microscopy (XE150, PSIA, Suwon-si, Republic of Korea) with a scan area of 10 × 10 µm^2^. The acquired AFM images were processed to evaluate surface roughness and crystalline particle distribution. Additionally, three-dimensional (3D) topographic images and height profiles were extracted to provide a detailed visualization of the film morphology.

The electrical characteristics of the devices, including current–voltage curves and breakdown voltages, were measured using a semiconductor characterization system (4200A-SCS, Keithley, Cleveland, OH, USA) connected to a microprobe station (APX-6B, WIT Co., Suwon, Republic of Korea), and capacitance–voltage curves were measured at frequencies of 1 MHz and 1 kHz using the same equipment. The measurement data were obtained by repeatedly measuring and taking the average value. The measurement system used has an error margin of approximately 3%. To evaluate device reliability, we examined the shift in the flat-band voltage (*V*_FB_) as a function of memory retention time and P/E cycle number. Memory retention was examined using a program pulse of 10 V for 1 s and an erase pulse of −10 V for 1 s. P/E cycling was performed using a pulse train of ±10 V and 10 ms.

## 3. Results and Discussion

### 3.1. Anantase TiO_2_ Films

**Figure 3:**
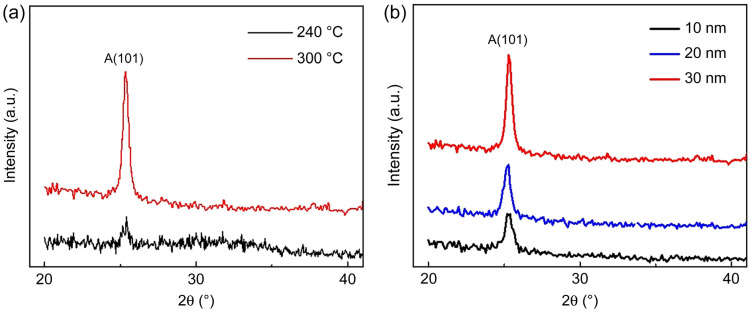
Effects of (**a**) deposition temperature and (**b**) film thickness on the X-ray diffraction patterns of TiO_2_ thin films deposited by remote plasma (RP) ALD.

To fabricate a semiconductor device with a TiO_2_ channel layer and HfO_2_ CTL, TiO_2_ was deposited using PE ALD and annealed to form the anatase phase. The X-ray diffraction patterns of the TiO_2_ films deposited at 240 and 300 °C (Figure 3a) showed that the former film was primarily amorphous and contained a small amount of anatase, whereas the latter film exhibited a highly crystalline anatase structure. The peak at 25.2° observed for the latter film correspond to the (101) plane of anatase [37,38]. The electrical conductivity of anatase is superior to that of rutile, which forms above 300 °C [32,34,38,56]. Therefore, for CTM device fabrication, the ALD of TiO_2_ was conducted at 300 °C. The X-ray diffraction patterns of the variable-thickness (10–30 nm) TiO_2_ films deposited at 300 °C (Figure 3b) revealed that the peak intensity increased with the increasing film thickness. For oxide-based MOSFETs, which are advantageous for low-power semiconductor devices, the channel layer should be as thin as possible while maintaining good crystallinity to ensure high mobility. Hence, the highly crystalline 30 nm thick TiO_2_ films were considered most suitable for our devices [30,35].

**Figure 4:**
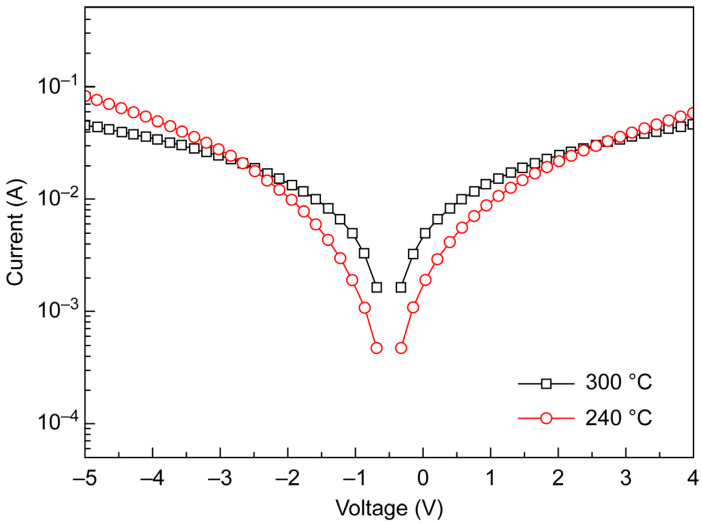
*I*–*V* curves of metal/TiO_2_/metal structures with 30 nm thick TiO_2_ films deposited by RP ALD.

The *I*–*V* curves of the 30 nm thick TiO_2_ thin films deposited at different temperatures were recorded using a metal/TiO_2_/metal structure (Figure 4). TiO_2_ deposited at 300 °C exhibited electrical properties superior to those of TiO_2_ deposited at 240 °C when an electric field was applied during semiconductor property measurements, which was ascribed to the formation of anatase in the former case.

### 3.2. CTM Devices with TiO_2_ and HfO_2_ Layers

**Figure 5:**
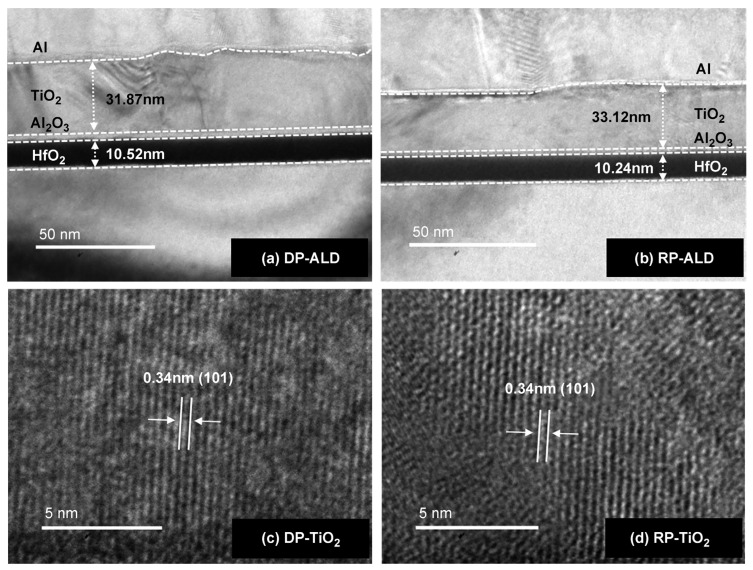
Cross-sectional transmission electron microscopy images of (**a**,**b**) devices and (**c**,**d**) TiO_2_ thin films fabricated by (**a**,**c**) DP and (**b**,**d**) RP ALD.

Figure 5 presents the cross-sectional transmission electron microscopy images of the semiconductor devices fabricated using DP and RP ALD. To prevent charge leakage from the HfO_2_ layer, a 2 nm thick Al_2_O_3_ tunneling layer was inserted between the HfO_2_ and TiO_2_ layers. The thicknesses of the HfO_2_ and TiO_2_ layers were set to approximately 10 and 30 nm, respectively, based on the results of previous studies [35,47]. No notable differences were observed between the devices fabricated using DP (Figure 5a) and RP (Figure 5b) methods, and a smooth well-defined interface between TiO_2_ and HfO_2_ without any reaction layers was observed in both cases. The thicknesses of the DP and RP HfO_2_ layers were 10.5 and 10.2 nm, respectively. Given the higher deposition rate of the DP method, 95 and 135 cycles were used for DP and RP ALD, respectively, with the respective growth per cycle values equaling 0.11 and 0.08 Å [57,58]. The DP and RP TiO_2_ layers had thicknesses of 31.9 and 33.1 nm, respectively. The (101) lattice spacings of DP (Figure 5c) and RP TiO_2_ (Figure 5d) were measured as 0.34 nm and agreed with the known d(101) spacing of anatase (~0.332 nm), indicating that the anatase phase was predominantly obtained. To further investigate the atomic structure differences between the two processes, chemical bonding analysis using X-ray photoelectron spectroscopy should be conducted [37,38,59].

**Figure 6:**
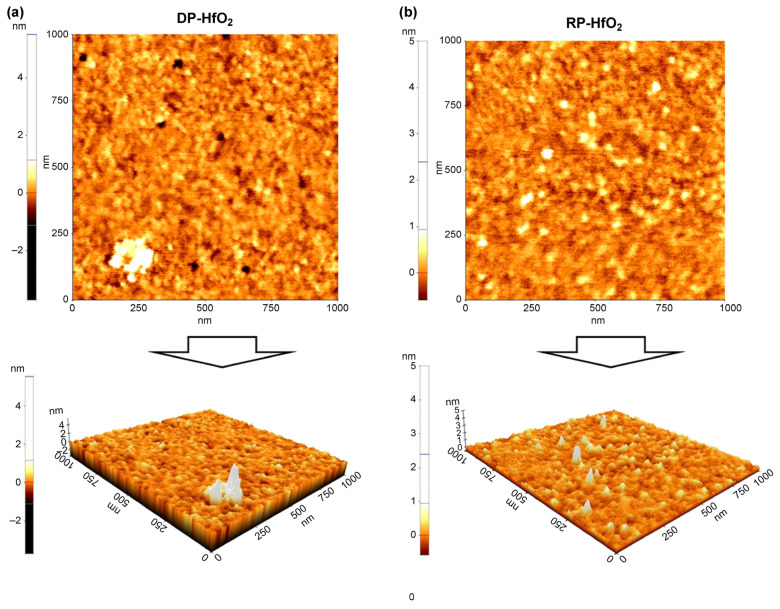
Atomic force microscopy images of (**a**) DP and (**b**) RP HfO_2_ thin films.

Considering the potential impact of plasma damage on film deposition and growth, we analyzed the surface morphology of the DP and RP HfO_2_ thin films by atomic force microscopy (Figure 6), revealing that these films had average surface roughness (Ra) values of 0.185 and 0.139 nm, respectively. Thus, a smaller and more uniform crystal size and, hence, smoother surface were observed in the latter case. Although the HfO_2_ film deposited at a high plasma power of 2600 W experienced minimal Ar-ion bombardment, it had a low Ra, probably because the increased distance between the substrate and plasma source reduced plasma-induced damage [39,42,46,50].

**Figure 7:**
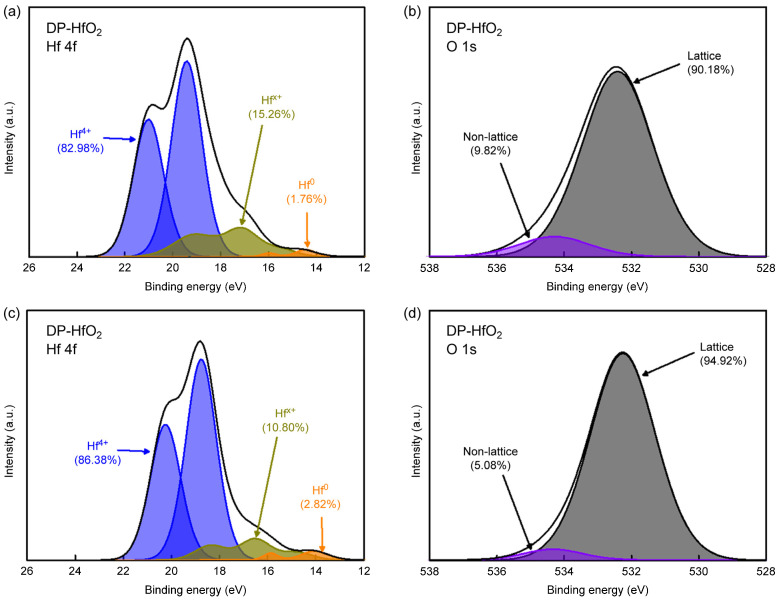
(**a**,**c**) Hf 4f and (**b**,**d**) O 1s spectra of (**a**,**b**) DP and (**c**,**d**) RP HfO_2_ films.

The trapping of charges in HfO_2_ thin films is attributed to intrinsic defects within HfO_2_, such as oxygen vacancies and interstitial oxygen atoms [18,20,26]. To compare the defect densities of the DP and RP HfO_2_ thin films, we determined their lattice-to-nonlattice-bond ratios from the corresponding Hf 4f spectra (Figure 7a,c, respectively), which were deconvoluted into the peaks of Hf^4+^, Hf^x+^, and Hf^0^. Each peak was split into 4f_5/2_ and 4f_7/2_ Gaussian signals with a 3:4 intensity ratio. The suboxide signal (Hf^x+^) associated with oxygen defects was obtained by subtracting the Hf^4+^ signal from the original signal. The fractions of nonstoichiometric oxygen defects (represented by Hf^x+^ and Hf^0^) in the DP and RP HfO_2_ thin films were calculated as 17.0% and 13.6%, respectively. Thus, the DP film contained more oxygen defects than the RP film [22,52].

Figure 7b,d show the deconvoluted O 1s spectra of the DP and RP HfO_2_ thin films, respectively, revealing the presence of lattice (oxygen directly bonded to Hf within the HfO_2_ crystal) and nonlattice (oxygen vacancies, O–H, and C–O moieties not directly bonded to Hf) oxygen. The fractions of the nonlattice peak, which represent defects, were calculated as 9.82% (DP) and 5.08% (RP). The higher content of nonstoichiometric hafnia and nonlattice oxygen in DP HfO_2_ indicates a greater number of intrinsic defects, which aligns with the defect content trend extracted from the Hf 4f spectra. These results agree with the breakdown voltage characteristics and long-term retention properties [26,60,61].

**Figure 8:**
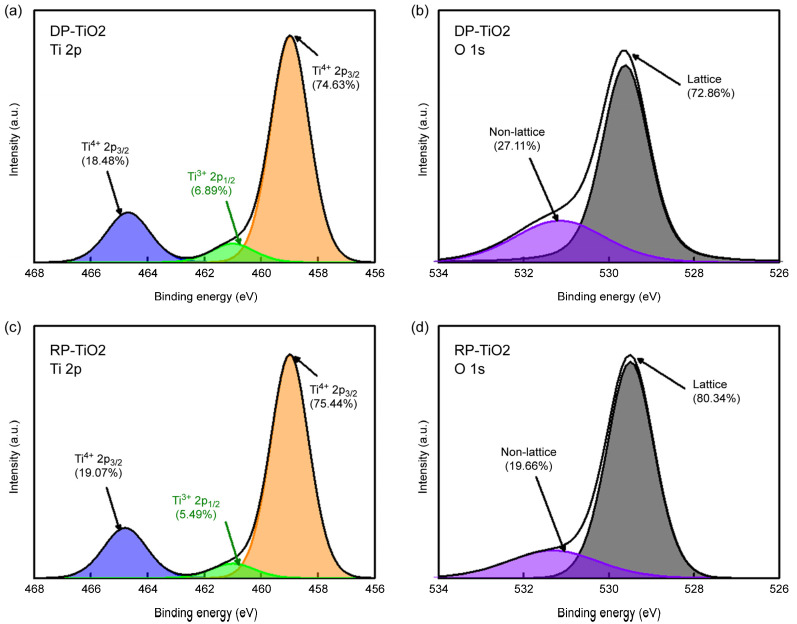
(**a**,**c**) Ti 2p and (**b**,**d**) O 1s spectra of (**a**,**b**) DP and (**c**,**d**) RP TiO_2_ films.

The 30 nm thick TiO_2_ films fabricated using DP and RP ALD were examined by high-resolution X-ray photoelectron spectroscopy (Figure 8). The doublet observed in the Ti 2p spectra, namely the Ti 2p_3/2_ (binding energy 458.6 eV) and Ti 2p_1/2_ (binding energy 464.4 eV) peaks, is due to spin–orbit coupling and indicates the presence of Ti^4+^ in the TiO_2_ lattice [51] (Figure 8a,c). The Ti 2p_1/2_ shoulder peak at 460.8 eV corresponds to Ti^3+^ in Ti_2_O_3_, indicating the copresence of TiO_2_ and Ti_2_O_3_ [62]. The change in the positions of these peaks upon going from DP to RP ALD suggests that the plasma conditions during deposition influence Ti phase formation and thus implies that some oxygen bonded to Ti ions is converted into vacancies or nonlattice oxygen. Upon going from DP to RP ALD, the area of the Ti^3+^ peak decreased from 6.89% to 5.49%, while the combined area of the Ti^4+^ peaks increased from 93.11% to 94.51%. The reduction in the Ti^3+^ peak area suggests that less Ti_2_O_3_ or nonlattice oxygen/vacancies were formed [63]. The O 1s spectra of the DP (Figure 8b) and RP TiO_2_ (Figure 8d) thin films were deconvoluted into the peaks of lattice oxygen and nonlattice oxygen at 529.9 and 531.3 eV, respectively [64,65]. In the case of DP TiO_2_, the relative areas of the lattice oxygen (529.7 eV) and nonlattice oxygen (531.5 eV) peaks were 72.86% and 27.11%, respectively. In the case of RP TiO_2_, the lattice (529.7 eV) and nonlattice (531.5 eV) oxygen peaks had relative areas of 80.34% and 19.66%, respectively. The higher contribution of the nonlattice oxygen peak observed for DP TiO_2_ suggests the enhanced formation of oxygen vacancies. This result is consistent with the Ti 2p spectra in Figure 7a,c, indicating that the application of RP ALD reduces the content of nonlattice oxygen and oxygen vacancies and thereby lowers the conductivity of TiO_2_, which may help reduce the loss current in the off state (*I*_off_) of the corresponding semiconductor devices [35,37,66,67,68].

### 3.3. Electrical Characteristics of MHOTM Devices

**Figure 9:**
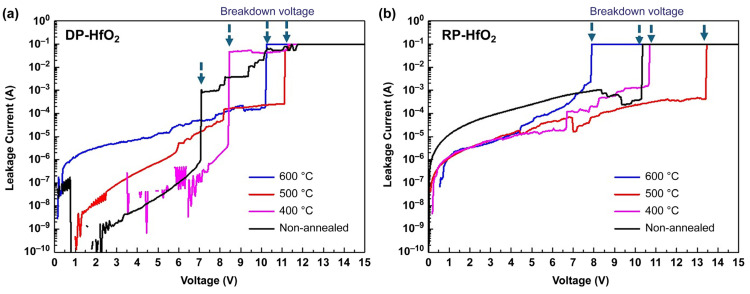
Leakage current–voltage plots of (**a**) DP and (**b**) RP HfO_2_ thin films prepared at different deposition temperatures.

The electric breakdown voltages of the 10 nm thick DP (Figure 9a) and RP HfO_2_ (Figure 9b) thin films annealed at different temperatures were determined by recording the current while increasing the voltage from 0 to 15 V in 0.05 V steps. The voltages determined for the DP films (9, 11, and 8 MV/cm at 400, 500, and 600 °C, respectively) were lower than those obtained for the RP films (11, 13, and 10 MV/cm at 400, 500, and 600 °C, respectively) by 1–2 MV/cm, which indirectly indicates that DP produced more internal defects than RP, resulting in a lower breakdown voltage. During DP ALD, plasma discharge occurs in the same chamber as deposition, directly impacting the surface of the thin film and generating defects, which play a vital role in breakdown performance degradation, especially at temperatures below 500 °C. These results are consistent with those of X-ray photoelectron spectroscopy analysis. Given that a 1 V difference in voltage can markedly affect the defect rate and lifetime of semiconductor devices, these findings are meaningful. Unannealed HfO_2_ thin films exhibited the lowest breakdown voltage because of their high concentration of oxygen vacancies. As the annealing temperature increased, the number of internal and interfacial defects, such as oxygen vacancies, decreased, and the breakdown voltage therefore increased. However, at temperatures exceeding 600 °C, the defect content decreased, and internal nanocrystallization began, leading to a leakage current increase and breakdown voltage decrease. Based on the reduced leakage current and highest breakdown voltage observed, the optimal annealing temperature for the RP HfO_2_ thin films was determined as 500 °C.

**Figure 10:**
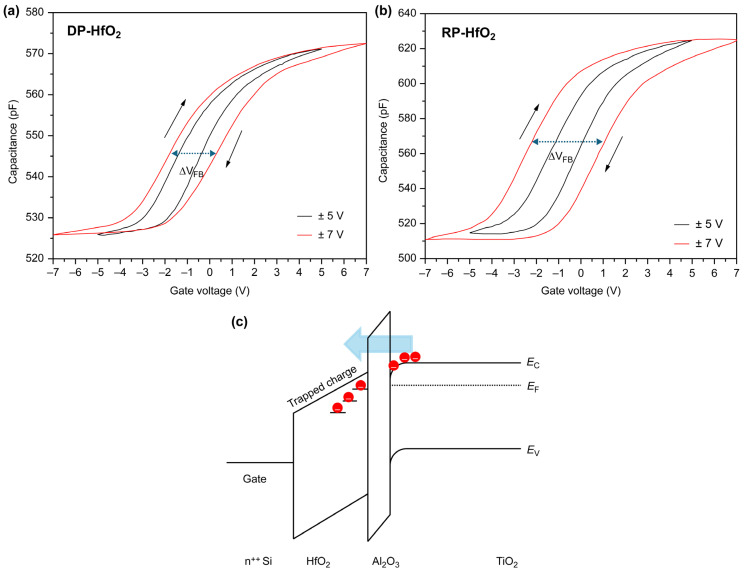
Capacitance–voltage curves of (**a**) DP HfO_2_- and (**b**) RP HfO_2_-based capacitors recorded at different sweeping voltages and (**c**) band diagram of the MHOTM capacitor indicating charge-trapping characteristics.

To analyze the interfacial damage and internal defects in thin films, we recorded the C–V curves of DP (Figure 10a) and RP (Figure 10b) HfO_2_-based capacitors with TiO_2_ channel layers in a forward–backward dual-sweeping fashion at room temperature and 1 MHz. When an electron is trapped at a charge-trapping site in an oxide film under a positive bias, V_FB_ shifts in the positive direction. However, when a hole is trapped under a negative bias and an electron is detrapped, this voltage shifts in the negative direction. ∆V_FB_ is defined as the difference between the V_FB_ values in the program and erase states and increases with the increasing sweeping voltage. For the DP HfO_2_-based capacitor, ∆V_FB_ minimally increased with an increase in the sweeping voltage. In contrast, for the RP HfO_2_-based device, ∆V_FB_ markedly increased with an increase in the sweeping voltage, reaching 3.5 V at a sweeping voltage of 7 V. This finding indicates the excellent charge-trapping efficiency and high potential of the RP HfO_2_ thin films for applications as CTLs [7,29]. Oxide thin films intended for use as CTLs should exhibit high charge-trap density (N_t_) values. Herein, the N_t_ values per unit area of the DP and RP HfO_2_ thin films were calculated at the point where the ∆V_FB_ saturated with an increase in the sweeping voltage [18,30]:

N_t_ = C_ox_ × ∆V_FB_/qA (1)

where C_ox_ is the capacitance in the accumulation region, q is the electron charge, and A is the effective area of the Al top electrode. The N_t_ values of the DP and RP HfO_2_ thin films at the sweeping voltage of ±7 V were determined as 2.84 × 10^13^ and 4.44 × 10^13^ cm^−2^, respectively. Notably, the value of the RP film was more than twice that of the DP film. Figure 10c shows the band diagram of the MHOTM device fabricated herein and illustrates the principle of charge storage. TiO_2_ exhibits n-type semiconductor properties, and under the application of a negative electric field, electrons move via tunneling through the Al_2_O_3_ layer, illustrating the principle of charge storage.

**Figure 11:**
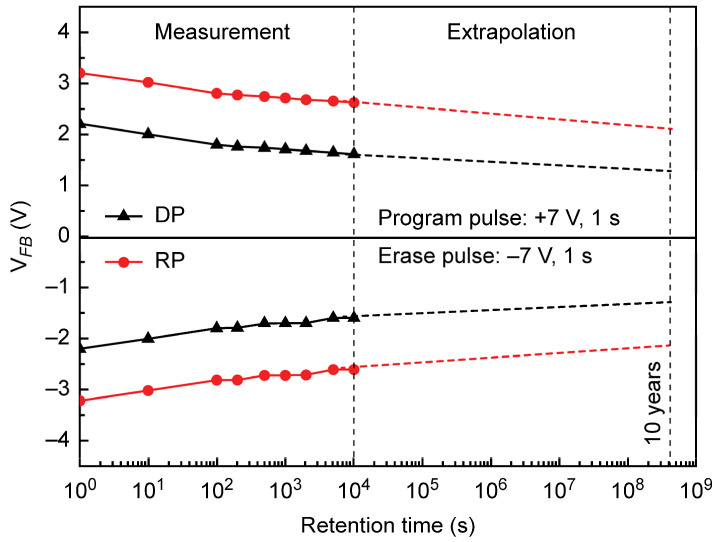
Memory retention characteristics of DP and RP HfO_2_–TiO_2_ CTM devices. The flat-band voltage (*V*_FB_) is plotted as a function of retention time, with data measured up to 10^4^ s and extrapolated for up to 10 years.

To evaluate the reliability of the DP and RP HfO_2_–TiO_2_ CTM devices, we measured *V*_FB_ as a function of memory retention time, revealing a negative correlation between the latter parameter and Δ*V*_FB_ (Figure 11). The voltage application conditions were as follows: program pulse of 7 V for 1 s and erase pulse of −7 V for 1 s. Memory retention times were plotted on a logarithmic scale, with measurements taken up to 10^4^ s, and *V*_FB_ values were extrapolated for up to 10 years. For the DP HfO_2_ CTM device, ∆*V*_FB_ decreased by 43% over 10 years (from 4.4 to 2.5 V), whereas the RP HfO_2_ CTM device exhibited a smaller decrease of 34% (from 6.4 to 4.2 V). The larger charge loss observed in the former device is attributed to interface defects between the tunneling oxide (Al_2_O_3_) and TiO_2_ layers, as well as to the promotion of charge detrapping by the charge-trapping sites within the tunneling oxide. However, even the RP HfO_2_ CTM device demonstrated a memory retention performance inferior to that of previously reported high-*k* oxide-based CTM devices. This is likely due to the absence of a blocking oxide layer, such as Al_2_O_3_ or SiO_2_, which is essential for preventing charge diffusion. Further structural optimization is expected to enable the fabrication of devices with improved ∆*V*_FB_ and memory retention characteristics.

## 4. Conclusions

Metal–charge trap–insulator–semiconductor–metal devices incorporating CTLs (HfO_2_ thin films), semiconductor channel layers (TiO_2_ thin films), and tunneling layers (Al_2_O_3_ thin films) were fabricated. The corresponding thin films were deposited using PE ALD. For the CTLs, RP and DP ALD were used, and the characteristics of the two semiconductor device types were compared. The influence of RP and DP processes on the charge-trapping properties of the HfO_2_ films and semiconductor characteristics of the TiO_2_ layers was systematically analyzed after annealing at temperatures ranging from 400 °C to 600 °C. For the TiO_2_ thin films, the anatase phase with excellent semiconductor properties was obtained by performing ALD at 300 °C. The memory characteristics of RP HfO_2_–TiO_2_ CTM devices were superior to those of DP HfO_2_–TiO_2_ CTM devices. The results of *C*–*V* measurements indicated that a change from DP to RP ALD resulted in an improved *V*_FB_ (by ~2 V) and increased breakdown voltage (by >2 V). These enhancements were attributed to the separation of plasma during the RP process, where only oxygen radicals were supplied to the chamber to minimize plasma-induced damage and contamination. This process also reduced the content of oxygen vacancies at the interface caused by Ar^+^ bombardment during deposition. Furthermore, the RP HfO_2_ thin film exhibited a high charge-trap density and low interface trap charge, which resulted in a superior breakdown voltage and wider memory window. These properties indicate that the RP HfO_2_ thin films are well suited for use in next-generation semiconductor synaptic devices.

## Data Availability

The original contributions presented in this study are included in the article. Further inquiries can be directed to the corresponding author.

## Abbreviations

The following abbreviations are used in this manuscript:

ALD	Atomic layer deposition
DP	Direct plasma
CTL	Charge-trapping layer
CTM	Charge-trapping memory
MHOTM	Sin++/HfO_2_/Al_2_O_3_/TiO_2_/Al
PE	Plasma-enhanced
P/E	Program/erase
RP	Remote plasma
